# Structural Adaptation and Heterogeneity of Normal and Tumor Microvascular Networks

**DOI:** 10.1371/journal.pcbi.1000394

**Published:** 2009-05-29

**Authors:** Axel R. Pries, Annemiek J. M. Cornelissen, Anoek A. Sloot, Marlene Hinkeldey, Matthew R. Dreher, Michael Höpfner, Mark W. Dewhirst, Timothy W. Secomb

**Affiliations:** 1 Department of Physiology, Charité, Berlin, Germany; 2 Deutsches Herzzentrum Berlin, Berlin, Germany; 3 Laboratoire Matière et Systèmes Complexes (MSC), UMR 7057 CNRS & Université Paris-Diderot, Paris, France; 4 Department of Man Machine Systems, Faculty of Mechanical Engineering and Marine Technology, Delft University of Technology, Delft, The Netherlands; 5 National Institutes of Health, Clinical Center, Radiology and Imaging Sciences, Bethesda, Maryland, United States of America; 6 Department of Radiation Oncology, Duke University Medical Center, Durham, North Carolina, United States of America; 7 Department of Physiology, University of Arizona, Tucson, Arizona, United States of America; University of Virginia, United States of America

## Abstract

Relative to normal tissues, tumor microcirculation exhibits high structural and functional heterogeneity leading to hypoxic regions and impairing treatment efficacy. Here, computational simulations of blood vessel structural adaptation are used to explore the hypothesis that abnormal adaptive responses to local hemodynamic and metabolic stimuli contribute to aberrant morphological and hemodynamic characteristics of tumor microcirculation. Topology, vascular diameter, length, and red blood cell velocity of normal mesenteric and tumor vascular networks were recorded by intravital microscopy. Computational models were used to estimate hemodynamics and oxygen distribution and to simulate vascular diameter adaptation in response to hemodynamic, metabolic and conducted stimuli. The assumed sensitivity to hemodynamic and conducted signals, the vascular growth tendency, and the random variability of vascular responses were altered to simulate ‘normal’ and ‘tumor’ adaptation modes. The heterogeneous properties of vascular networks were characterized by diameter mismatch at vascular branch points (d^3^_var_) and deficit of oxygen delivery relative to demand (O_2def_). In the tumor, d^3^_var_ and O_2def_ were higher (0.404 and 0.182) than in normal networks (0.278 and 0.099). Simulated remodeling of the tumor network with ‘normal’ parameters gave low values (0.288 and 0.099). Conversely, normal networks attained tumor-like characteristics (0.41 and 0.179) upon adaptation with ‘tumor’ parameters, including low conducted sensitivity, increased growth tendency, and elevated random biological variability. It is concluded that the deviant properties of tumor microcirculation may result largely from defective structural adaptation, including strongly reduced responses to conducted stimuli.

## Author Summary

Blood vessels of tumors have abnormal structures, being irregular and tortuous. Oxygen supply to tumors is heterogeneous, with regions of low oxygen that resist radiation treatment and some types of chemotherapy. Blood vessels undergo continual structural change (adaptation) in response to blood flow and metabolite levels. Our hypothesis is that abnormal adaptation of tumor microvessels causes their heterogeneous structure and impaired function. We used computational models to estimate blood flow and oxygen delivery and to simulate diameter adaptation in networks of microvessels, using network structures derived from microscopic observations of living normal and tumor tissues. The simulation of adaptation depends on several parameters that describe vessel sensitivity to fluid shear stress, to blood pressure, to oxygen levels, and to signals propagated along vessel walls (conducted response). We found that structural adaptation of a tumor network using parameters derived from normal tissues could ‘normalize’ the network, giving it properties similar to a normal tissue. Conversely, adaptation of normal networks using parameters derived from the tumor network, including reduced conducted response, gave tumor-like properties. We conclude that the deviant properties of tumor microcirculation may result largely from defective structural adaptation, including reduced conducted responses.

## Introduction

Solid tumors require an internal vasculature in order to grow beyond a few mm in size and to metastasize [1]. Tumor vascular networks are known to be fundamentally different from normal vasculature (e.g. [2]–[4]). Vessels are typically immature, tortuous, dilated, and hyperpermeable and network structures are spatially and temporally heterogeneous, resulting in regions of hypoxia within the tumors [5],[6]. The characteristics of tumor microvasculature are important not only for the growth and development of tumors, but also for the efficacy of chemo- and radiotherapeutic treatment [4]. Delivery of cytotoxic agents via the blood circulation is impaired due to the heterogeneous blood flow distribution [2],[7]. Many treatment modalities are based on the higher susceptibility of the fast dividing tumor cells, whereas cells in hypoxic regions exhibit a low tendency to undergo mitosis, and may not be exposed to effective concentrations of chemotherapeutic substances. Susceptibility to radiation treatment is diminished in hypoxic regions, because lack of oxygen reduces the production of toxic free radicals. Furthermore, hypoxic areas may lead to clonal selection of highly malignant tumor cells that have lost sensitivity to apoptosis [8] or that have a drug-resistant phenotype [9].

These observations have stimulated two different concepts of vessel targeting in tumor treatment, which may be synergistic [10] or antagonistic [11]. On one hand, anti-angiogenic substances could be used to destroy tumor vasculature and consequently lead to the degradation of the majority of tumor cells, which depend on those vessels for their supply of oxygen and nutrients [12],[13]. However, tumor vascular networks often exhibit equal or even higher fractional intravascular volumes than normal tissues [14], showing that poor tumor oxygenation is not primarily related to a limited amount of vessels within the tumor, but rather to deficient structure of the vascular networks. Therefore, it was proposed that ‘normalization’ of tumor microvasculature could improve the delivery of oxygen and drugs to tumor cells and thus increase the susceptibility to cytotoxic anti-tumor agents or radiation in a combination therapy approach [2],[15],[16]. It was suggested that ‘normalization’ is a property of some effective anti-angiogenic agents, such as the VEGF-neutralizing monoclonal antibody bevacizumab and VEGF- receptor-specific tyrosine kinase inhibitors (sunitinib, vatalanib) [17],[18]. Such clinically established substances may restore the impaired balance of angiogenic stimulators and inhibitors in pathological angiogenesis, thereby leading to a normalized tumor microvasculature. However, the mechanisms involved in these processes remain to be elucidated as a basis for the development of additional approaches for normalizing tumor microvessel networks. Therefore, we here used computational simulations based on structural information obtained by intravital microscopy to identify factors that may account for the deficient structure of tumor microvascular networks.

**Figure 1:**
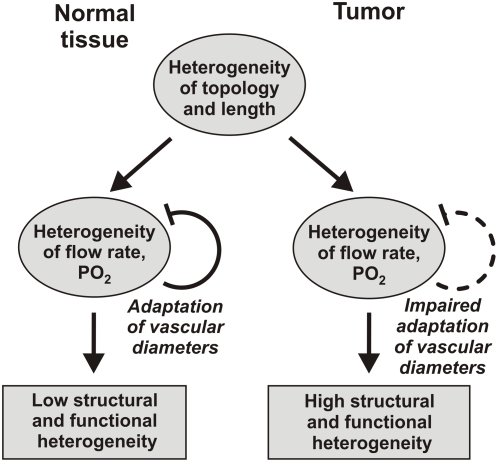
Conceptual basis of the study. Blunt headed arrows denote negative feedback loop, in which structural adaptation reduces heterogeneity in flow and oxygenation that is generated by heterogeneous vessel topology and lengths. The hypothesis is that this feedback loop is weaker (dashed curve) in tumors than in normal tissues. See text for further explanation.

The conceptual basis of the present study is illustrated in Figure 1. In all tissues, microvascular networks exhibit a degree of irregularity with respect to topological arrangement and lengths of vessel segments, as a consequence of the stochastic nature of angiogenesis and of the geometric constraints for space-filling vascular trees. The structural heterogeneity in turn leads to heterogeneous distributions of functional parameters, including local perfusion and oxygen partial pressure (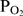). In normal tissues, structural adaptation of vessel diameters in response to local hemodynamic and metabolic stimuli reduces the heterogeneity to a low level, compatible with tissue functional requirements. The hypothesis of the present study is that impaired structural adaptation is a major cause for the high structural and functional heterogeneity observed in tumor networks.

## Results

**Figure 2:**
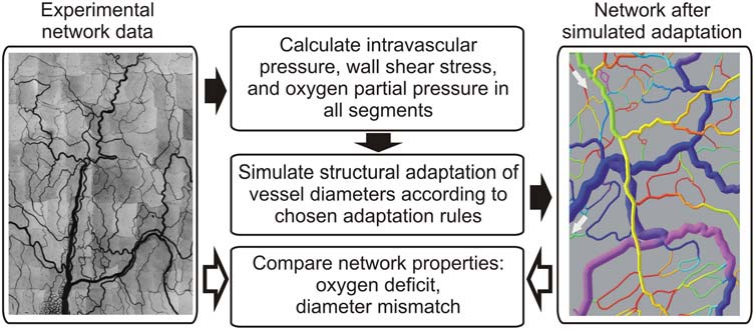
Summary of computational approach. In-vivo experimental data on network structure (vessel diameter, length and connection pattern) were acquired during intravital microscopy (photomontage, left panel). Blood flow and oxygen distribution were calculated and diameter adaptation was simulated until a steady state was reached (computer visualization, right panel). Structural and functional parameters after simulated adaptation with different adaptation rules were compared with corresponding parameters for tumor and mesentery networks with measured diameters.

The computational approach is summarized in Figure 2. Data describing the angioarchitecture of a tumor microvascular network and three control (mesentery) networks were used to calculate blood flow, wall shear stress, pressure and 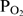 for all vessel segments. These parameters were considered as stimuli in the simulation of structural adaptation of vessel diameters, which was carried out using previously established rules for normal vascular networks [19] and with rules modified to achieve tumor-like network characteristics. Here, the term ‘rules’ refers to quantitative relationships between the stimuli acting on a segment vessel and the resulting structural diameter changes. Network properties resulting from simulated diameter adaptation were compared with properties corresponding to vessel diameters as measured experimentally.

**Figure 3:**
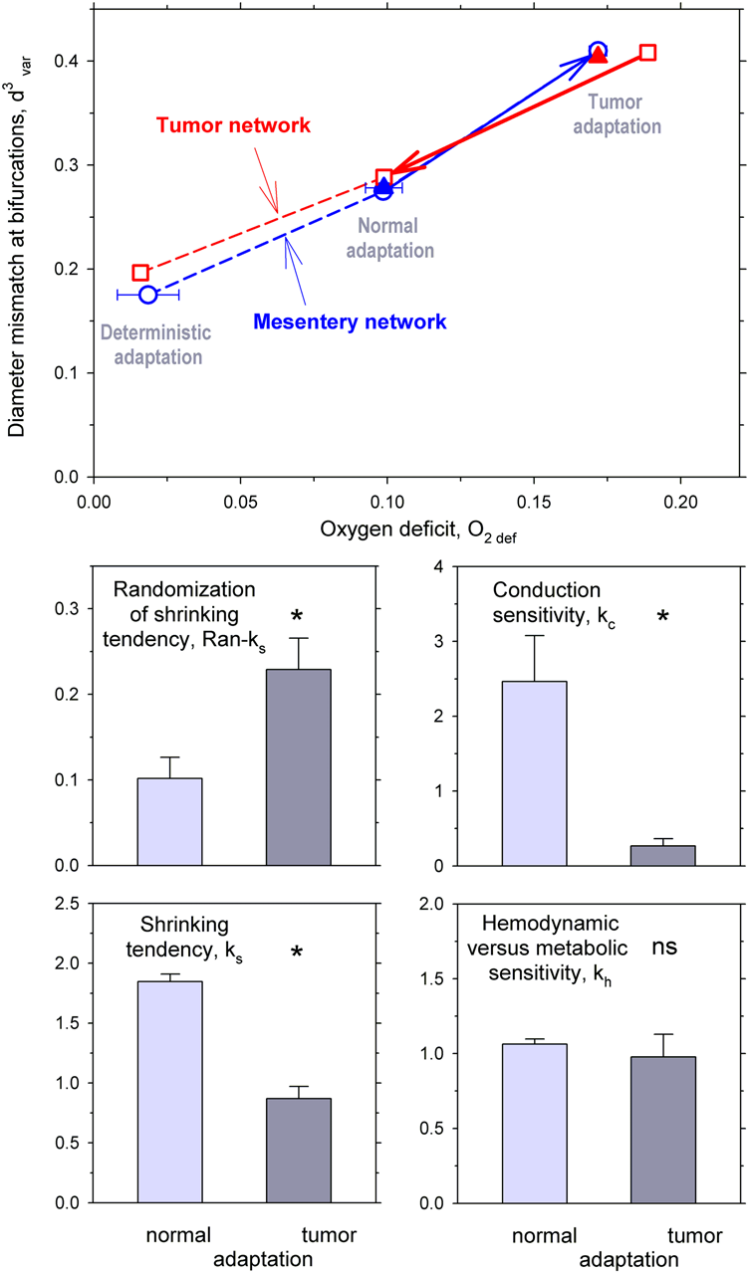
Network characteristics and sensitivity parameters for simulated normal and tumor modes of vascular adaptation. *Top panel:* Dependence of oxygen deficit and diameter mismatch on assumed adaptation mode. Filled triangles: results for experimentally measured vessel diameters for tumor network (red) and mesenteric networks (blue, mean with standard deviation). Open symbols: predicted results using vessel diameters obtained with normal, tumor or deterministic adaptation models for the tumor network (red squares) and for mesenteric networks (blue circles). When the tumor network is subjected to normal adaptation (red arrow), the resulting characteristics are close to those of mesentery networks with measured diameters. Conversely, when the mesentery networks are subject to tumor adaptation (blue arrow), they achieve tumor-like characteristics. If deterministic adaptation is assumed, low values for O_2def_ and d^3^_var_ are obtained which do not differ significantly for tumor and mesenteric networks. *Lower panels:* Sensitivity parameters for simulated diameter adaptation of mesenteric networks. Predicted parameter values for the optimization to characteristic network properties (O_2def_, d^3^_var_, V_tot_, ΔP) as obtained with measured vessel diameters for the mesenteric networks (‘normal adaptation’, left) are compared to those obtained for tumor networks (‘tumor adaptation’, right). Mean data for three networks are shown with standard deviations. Significant differences are found for Ran-k_s_, k_c_, and k_s_ but not for k_h_.

Two parameters were used to characterize network properties (see *Methods*). Structural irregularity was described by the diameter mismatch at bifurcations, d^3^_var_, and functional state was described by the oxygen deficit, O_2def_. The upper panel of Figure 3 shows values of O_2def_ and d^3^_var_ describing functional status and structural heterogeneity of microvessel networks, calculated from measured diameters and from diameters predicted by simulated structural adaptation. When measured diameters are used (filled triangles), both O_2def_ and d^3^_var_ are higher for tumor than for normal networks. This reflects the known irregularity and functional deficiency of tumor microvasculature.

The simulated adaptation is controlled by several sensitivity parameters: k_h_, the hemodynamic sensitivity relative to the metabolic sensitivity; k_p_ the sensitivity to pressure; k_c_ the conduction sensitivity; k_s_ a basal vascular tendency to reduce vessel diameter in absence of growth stimuli (‘shrinking tendency’); Ran-k_s_, a parameter describing the randomness of the biological reaction. These parameters were varied to simulate the effects of different adaptation modes. For the results obtained by simulated structural adaptation (open symbols), the values of k_h_, k_c_, k_s_, Ran-k_s_ were optimized to match normal or tumor characteristics (see *Methods*). These results demonstrate that the levels of O_2def_ and d^3^_var_ are dependent on the assumed adaptation mode. Use of the ‘tumor adaptation’ mode leads to high structural heterogeneity and oxygen deficits, irrespective of the network structure used. In contrast, ‘normal adaptation’ leads to O_2def_ and d^3^_var_ values close to those obtained with experimentally measured vessel diameters for the mesentery. These results support the hypothesis that the functional deficits of tumor microcirculation are largely due to impaired vascular diameter adaptation.

For comparison purposes, corresponding results were also obtained with Ran-k_s_ set to zero, a case referred to as ‘deterministic’ adaptation. Values of O_2def_ and d^3^_var_ are then reduced in both tumor and mesentery networks. The results in this case, including a near zero level of oxygen deficit, may be more representative of the *in-vivo* situation in normal networks than the results for ‘normal’ adaptation, since the influence of experimental diameter measurement errors is abolished by deterministic adaptation.

**Figure 4:**
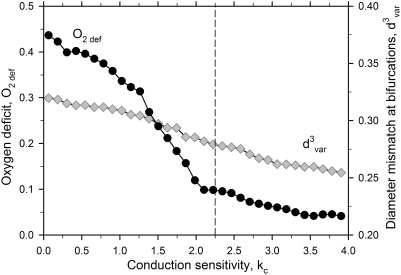
Predicted effects of signals conducted along the vessel wall on network characteristics. For a mesenteric network with 546 vessel segments, the conduction sensitivity was varied around the value used for ‘normal adaptation’ (vertical dashed line). Reduction of conduction strength below this level leads to a small increase in diameter mismatch and to development of a pronounced oxygen deficit despite a fixed level of bulk tissue perfusion.

The lower panels of Figure 3 give the optimized adaptation sensitivity parameters corresponding to ‘normal’ and ‘tumor’ adaptation. Relative to normal adaptation, tumor adaptation requires increased biological variability (higher Ran-k_s_), nearly abolished conduction (very low k_c_) and increased vascular growth tendency (lower shrinking tendency, k_s_). The balance between hemodynamic and metabolic sensitivity (k_h_) is not significantly different in the two cases. The importance of the conducted signal for the difference between normal and tumor adaptation was further explored by varying the conduction parameter k_c_. As shown in Figure 4, a reduction of k_c_ leads to a small increase in diameter mismatch and a substantial increase in oxygen deficit.

**Figure 5:**
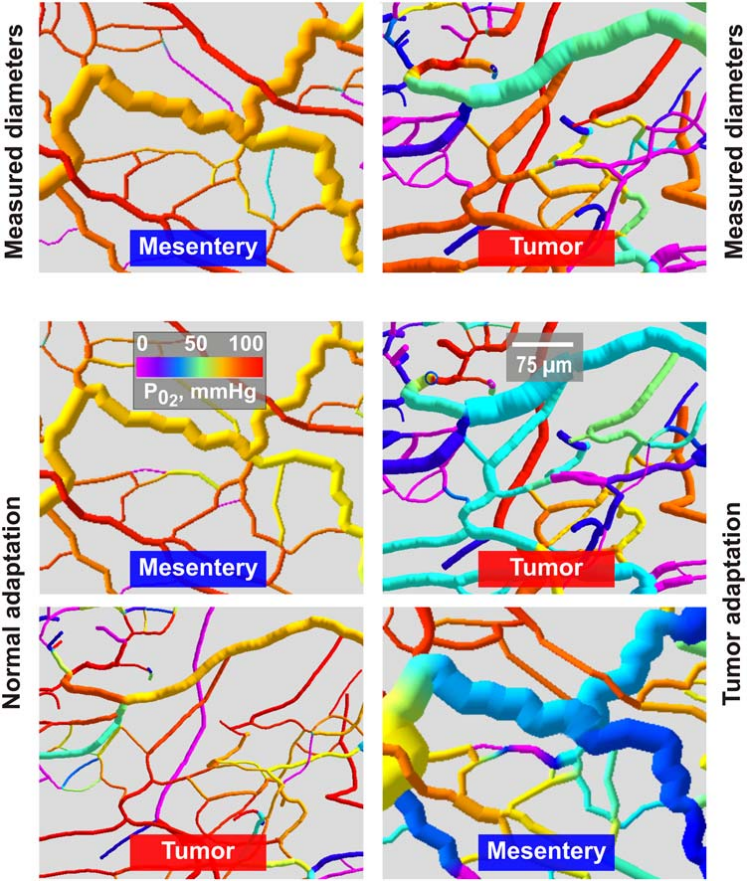
Computer visualizations of a mesenteric network and the tumor network color coded for 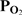. Top row: results obtained with measured vessel diameters. Middle and bottom rows: simulated ‘normal adaptation’ and ‘tumor adaptation’. The tumor network with measured diameters and either network subjected to ‘tumor adaptation’ (right column) exhibit high structural heterogeneity and uneven flow distribution in comparison to the mesentery network and either network subjected to ‘normal adaptation’ (left column). For the adapted networks, results of individual randomized runs are shown. Values of oxygen deficit (O_2def_) and diameter mismatch (d^3^_var_) were similar to the mean values reported in Figure 3. For the sake of a better representation of individual vessel segments, only parts of the simulated networks are shown. The mesentery network comprised a total of 546 vessels supplying an area of about 4×7 mm, whereas the tumor vascular network comprised a total of 290 vessels in an area of about 1×0.8 mm.

The distributions of vessel 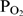 in regions of a mesenteric and the tumor network are shown in Figure 5. When subjected to ‘normal adaptation’, both networks exhibit close similarities to the experimentally observed mesenteric network, with diameters varying smoothly along flow pathways and high levels of 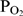. In contrast, ‘tumor adaptation’ leads to substantial irregularity in both networks, including regions with very low values of 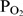.

## Discussion

In recent years, a number of studies have used mathematical models to analyze the development of tumor vasculature and its relation to tumor growth [20]–[24]. The modeling approach used here differs from those studies. It focuses not on the generation of new vessels by angiogenesis or vasculogenesis, but rather on the structural adaptation of existing vessels [25] corresponding to vascular maturation or remodeling. In two previous studies [26],[27], a similar approach was applied to tumor vasculature. Structural adaptation of microvessel networks with predefined hexagonal geometries was simulated using the model of Pries et al. [28], and effects of excluding or including the various stimuli were examined. Based on qualitative features of the resulting network structures and oxygen distributions, those authors proposed that a lack of upstream and downstream information transfer may account for observed abnormal features of tumor vasculature.

The aim of the present study was to identify key parameters accounting for the defective diameter adaptation and functional behavior of existing tumor microvasculature. Therefore, a tumor microvessel network and normal control networks were visualized, reconstructed and analyzed. A mathematical model was used to estimate network blood flow and oxygen uptake and thereby to analyze hemodynamic and functional characteristics of normal and tumor networks. The main result of the present study is that deficiencies in the diameter adaptation of the existing vessels of a tumor network can largely account for the heterogeneity in oxygen supply and the generation of hypoxic regions in tumor vascular beds, which have negative implications for tumor treatment.

The available data did not allow for precise estimation of the parameters describing vascular adaptation in tumors. Reconstructions of larger and more complete tumor networks would be needed and the results obtained would likely differ among tumor types. However, increased structural heterogeneity and occurrence of hypoxic regions are typical features of many tumors [6],[29],[30] and the general trends in adaptive reactions are probably very similar to those presented here. Thus, malfunction of angioadaptation may be a general phenomenon of tumor vasculature, regardless of the tumor type, host tissue or tumor localization and despite quantitative differences in the vascularization between different tumor types (e.g. hypervascularized hepatocellular carcinoma as compared to hypovascularized cholangiocarcinoma) [31].

The present approach does not address questions related to vessel number and density or to the topological and three-dimensional structure of tumor vascular networks. These characteristics exhibit significant differences between tumor and normal networks and have obvious implications for tumor development and metastasis [32]. However, adaptation of vessel diameters is a potent mechanism to regulate relevant hemodynamic and functional properties of vascular beds independent of vascular density or architecture. The dynamic control of vessel diameters requires continuous adaptation to functional hemodynamic and metabolic signals acting in interrelated feedback loops [33]. Even small deviations of vessel diameter lead to substantial changes in flow resistance, blood flow distribution, intravascular hematocrit and oxygen delivery, as a result of the inverse fourth power dependence of flow resistance on diameter. Establishment of functionally adequate network properties, despite heterogeneities of network architecture leading to arterio-venous flow pathways of different length, requires tight control of vessel diameters [33]–[35]. Even relatively moderate deficiencies in vascular adaptive responses can lead to significant functional impairments.

**Figure 6:**
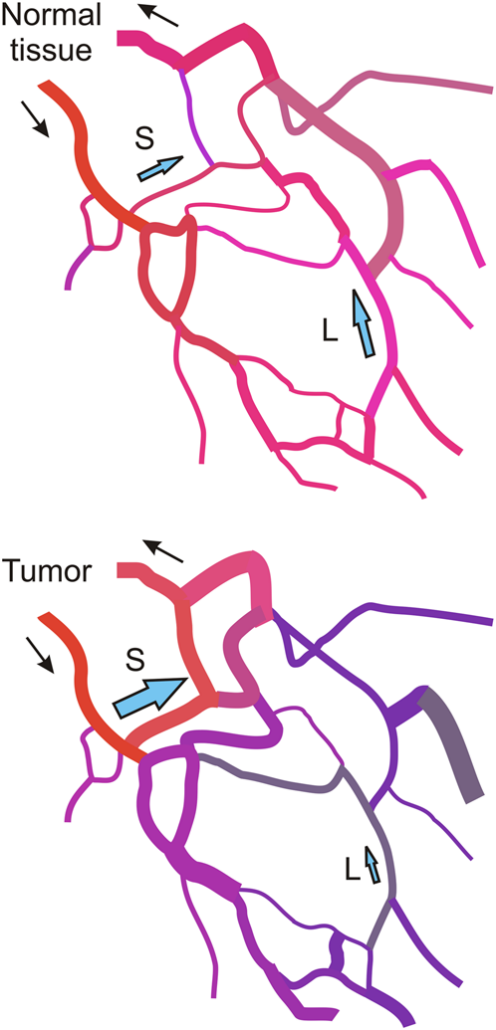
Schematic illustration showing hypothesized effect of information transfer on flow and oxygenation in networks. Top: in normal tissue, information transfer ensures that long pathways (L) receive adequate flow and short pathways (S) are not overperfused. Bottom: in tumor tissue, loss of conducted responses causes functional shunting via short pathways (S). Long pathways (L) are underperfused and hypoxic. Red and blue colors denote high and low oxygen levels respectively.

An important implication of this study is that strongly reduced transfer of information along vessels is a major cause for the observed deficiencies in the structure and function of tumor networks. In previous studies, we showed that information transfer upstream and downstream along blood vessels is essential for the formation of network structures with normal functional properties [19],[28]. In particular, such information transfer is needed to achieve a balance between flows in short and long flow pathways within a given network. In the absence of information transfer, flow increases in short pathways at the expense of longer pathways, leading to functional shunting of blood flow (Figure 6). The effect on red blood cell flux is even more pronounced, as a result of uneven red blood cell partition in diverging microvascular bifurcations. In consequence, regions supplied by longer pathways are inadequately supplied and tend to become hypoxic. Such behavior is consistent with observed properties of tumor vasculature, in which hypoxic regions are found even in the presence of relatively high levels of vascularity and perfusion.

To coordinate blood flow, the endothelial cells and smooth muscle cells of normal vessels communicate by conducting electrical signals via gap junctions [36]–[38]. By contrast, the walls of tumor microvessels in general do not exhibit a regular composition with endothelial cells, basement membrane and smooth muscle layer [39],[40]. Often these vessels are composed only of endothelial cells with altered ultrastructure or even of tumor cells [41]. Thus, it is conceivable that the conduction of electrical signals is substantially disturbed in tumors.

The importance of information transfer from smaller to larger vessels for adequate control of perfusion has been known for many decades. Segal and Duling identified the conduction of cellular electrical potentials along cells in the vascular wall as one important component of this reaction [42]. More recently, vascular connexins were identified as critical molecular components establishing gap junctions between vascular cells and thus allowing this type of electrical communication [43]–[45]. Gap junctions composed of the vascular connexins 38, 40 and 43 transmit changes in membrane potential between endothelial cells or between smooth muscle cells, and to a lesser degree between endothelial and smooth muscle cells.

In normal tissues, connexin expression may be modified by inhibition of the angiotensin converting enzyme or by changed shear stress at the endothelial surface. It was demonstrated [46] that VEGF induces disruption of endothelial gap junction communication. Upon VEGF treatment, a rapid and transient loss of endothelial cell-cell communication was observed, which involved VEGFR-2-driven activation of the ERK-family of MAP kinases to downregulate the phosphorylation of the gap junction forming connexin CX43. The development of chaotic tumor microvasculature during VEGF-induced angiogenic processes is thus likely to be based on a dysregulated communication via gap junctions of vessel forming endothelial cells. An interesting interpretation of the mechanism underlying normalization therapy with anti-VEGF treatment may be the restoration of the defective endothelial gap junction communication of existing vessels by inhibition of the tumor VEGF/VEGFR system. Future studies, focusing on expression and function of vascular connexins in tumors, will be needed to provide *in vivo* evidence for this hypothetical mechanism.

## Methods

### Experimental observations and reconstruction of vascular networks

All animal experiments were performed according to the applicable rules and regulations after obtaining approval by the University and State authorities for animal welfare. For dorsal skin fold window chamber experiments, nude mice (Balb/c *nu/nu*) were anesthetized with a cocktail of Ketamine and Xylazine (10∶1 w/w, 100 mg/kg Ketamine, IP) and prepared for window chamber implantation using common surgical techniques. A 1-cm diameter circular incision was made in a dorsal skin fold, over which a titanium chamber was surgically implanted. A single cell suspension of human squamous cell carcinoma (FaDu) cells was injected into the opposing fold of skin (1×10^4^ cells). A circular glass cover slip was placed over the incision through which the tumor and its associated vasculature were later visualized. After 10 days, 2 MDa fluorescein dextran (Invitrogen, Carlsbad CA) was injected intravenously and confocal imaging (LSM 510, Zeiss, Thornwood NY) of the vasculature immediately began. The resulting images were analyzed off-line using an application developed as an extension to the commercially available visualization and geometry reconstruction system AmiraDev [47]. For measurements of red blood cell velocity, DiI fluorescently labeled red blood cells were injected intravenously and the vascular network was epi-fluorescently imaged with a monochromatic SIT camera (Hamamatsu C2400-08) and recorded in S-VHS format (Mitsubishi BV-1000).

Mesenteric vascular networks of three male wistar rats prepared for intravital microscopy were observed and analyzed as described previously [48]. The small bowel was exteriorized, and fat-free portions of the mesentery selected for investigation. Papaverine (10^−4^ M) was continuously applied to suppress active vessel tone. Microvascular networks were scanned and video-recorded. From the video recordings, diameter and length were measured in all segments between branch points using a digital image analysis system. The topological and spatial arrangement of segments was determined. Hematocrit values and centerline velocities for vessels entering and leaving the network were measured with a digital image analysis system [49]. Centerline velocity was converted into mean blood velocity using a previously derived empirical relationship [50].

### Calculation of network hemodynamics and oxygen distribution

For a microvascular network with a given structure, i.e. a given arrangement of vessel segments with given lengths and diameters, the procedure to calculate hemodynamic parameters (blood flow, wall shear stress, blood pressure) and 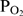 for all vessel segments has been described earlier [33],[51]. Information on connectivity, diameter and length of the vessels and boundary conditions of tumor and normal vascular networks was used as input to the hemodynamic model. For the mesentery networks, the boundary conditions comprise volume flow rates derived from velocity measurements and hematocrit for all segments feeding the network and volume flow rates for the vessels leaving the network, with the exception of the main venular draining vessel which was assigned a pressure of 13.8 mmHg to provide a fixed pressure reference. For the tumor network, the red blood cells were more difficult to track because of the opacity of the tumor tissue, and centerline velocity could not be determined for all boundary segments. For the remaining segments, blood cell velocity classes (slow, medium, and fast) were assigned from visual inspection of the video recordings. Estimates for the medium velocities were assigned using linear least-squares regression between velocity and diameter established for the vessel segments in which velocities could be measured. Slow and fast velocities were assigned according to the lower and higher boundaries of the 80% prediction interval of these measured data.

For given values of diameter, length and apparent blood viscosity in each vessel segment, volume flow rates and nodal (junction of segments) pressures were calculated by iteratively solving a system of linear equations [51]. Using the resulting volume flow rates, the hematocrit distribution was updated using a parametric description of red cell distribution at divergent bifurcations (phase separation effect) based on experimental findings in vivo [33],[52]. The resulting hematocrit values together with vessel diameters were then used to estimate local effective blood viscosity values (Fahraeus-Lindqvist effect) according to a parametric description of blood rheology in microvessels [53]. Estimations of flow distribution and local hematocrit and viscosity were iterated until convergence.

In the simplified model for oxygen transport used, oxygen is assumed to diffuse out of each vessel segment at a fixed rate per unit vessel length until the saturation drops to zero, neglecting possible effects of spatial vascular arrangement and diffusive crosstalk between vessels. Each vascular cross section was assumed to supply a tissue slice of 4000 µm^2^. In the experimental data, vessel spacing for all networks was similar (tumor: 4612 µm^3^ tissue/µm vessel; mesentery: 3673 µm^3^ tissue/µm vessel assuming a tissue thickness of 20 µm). With a consumption rate of 0.01 cm^3^O_2_/(cm^3^×min) typical for connective tissue, this corresponds to a consumption per unit vessel length and time of 4×10^−11^ cm^3^O_2_/(µm×min). These values were used throughout for mesentery and tumor networks to allow a direct comparison of consequences of structural differences between tumor and healthy vascular morphology on oxygen deficit. Oxygen consumption may be higher in tumor tissue than in healthy connective tissue. Incorporation of this difference would increase the oxygen deficit differences between tumor and control networks.

The convective influx of oxygen to a vessel segment was calculated as 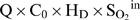, where Q is the flow rate, C_0_ = 0.5 cm^3^O_2_/cm^3^ is the oxygen binding capacity of red blood cells, H_D_ is the flow fraction of red cells (discharge hematocrit) and 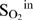 is the fractional oxygen saturation in the inflow to the segment. Average vascular oxygen saturation (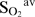) was calculated from 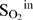 and the oxygen consumption in the vascular segment. The Hill equation was used to estimate average oxygen partial pressure (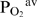) for each vessel segment as 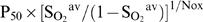 where P_50_ = 38 mmHg and N_OX_ = 3 according to experimental data for rat blood. Saturation of blood entering the networks was assumed to be 0.94 for the mesentery. For the tumor network, this approach was not applicable due to its smaller size and only the vessel segment with the highest flow was assigned the maximum 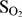. For other inflow segments with a flow above 5 nl/min, 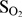 was calculated as the average of 3 internal vessel segments with similar size. For the remaining smallest boundary segments, 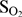 was set to 0.1.

### Network characteristics

In order to compare tumor and normal microvascular networks, parameters characterizing structural and functional network state were estimated. The structural irregularity of a network was described by the diameter mismatch at bifurcations, defined as the root mean square (RMS) value over all bifurcations (N) within the network of the difference between the cubed diameter of the mother segment (m) and sum of the cubed daughter segment diameters (d1, d2):
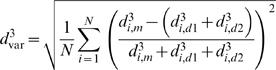
(1)The third power of diameter was chosen according to Murray's optimality concept [54], that the overall ‘cost’ of blood flow is minimal if flow is proportional to the cube of diameter, which implies that the sum of the cubed daughter vessel diameters equals the cubed mother vessel diameter at each bifurcation, i.e. d^3^_var_ = 0. This condition is approximately satisfied in normal arteriolar networks. The functional state of the network is described by the global oxygen deficit:
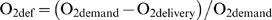
(2)where O_2demand_ is the oxygen demand of the entire network calculated as the product of the assumed oxygen consumption rate times the aggregate length of all segments within the network and O_2delivery_ is the difference in summed oxygen flow between inflow and outflow segments. In addition, the total intravascular volume of the network (V_tot_) and the pressure drop between feeding and draining segments (ΔP) were calculated. Since the network has multiple inflows and outflows, the flow-weighted average of the pressure drops along the flow pathways through the network was used.

### Simulation of vascular diameter adaptation

Microvessels continuously adjust their diameters in response to hemodynamic and metabolic stimuli [19],[55]. Here, a computational simulation was used to predict diameters of all vessel segments in the investigated networks, resulting from this structural adaptation. In accordance with experimental observations, the stimuli considered included local hemodynamic and metabolic parameters (blood pressure, wall shear stress, 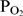). In addition to the local effects, the model includes convection of a metabolic signal substance downstream and conduction of signals along the vessel wall in the upstream direction. Conduction of electrical signals along vessel walls [37],[38] is an established mechanism in the regulation of vascular smooth muscle tone and seems also to be relevant for structural adaptation [19].

In the simulation [19], the diameter (D) of each segment in the network is assumed to vary according to a net stimulus (S_tot_) according to
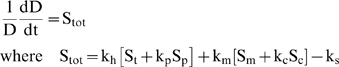
(3)Here, the constants k_h_, k_p_, k_m_ and k_c_ represent the sensitivities of vascular adaptation to various types of stimuli, as described below, and k_s_ represents a basal tendency of vessels to shrink in the absence of positive growth stimuli (‘shrinking tendency’). In the absence of information on the time course of adaptation, only the ratios of the values of the constants k_h_, k_m_ and k_s_ can be estimated, and not their absolute values, so k_h_ was set to 1 initially. The first term in S_tot_ represents responses to hemodynamic forces. S_t_ is calculated from wall shear stress, τ_w_, as log[τ_w_], and S_p_ is calculated from transmural pressure, P, as log[τ_e_(P)] where τ_e_(P) = 100−86 exp[−5000(log(log P))^5.4^] describes the shear stress set point as a function of P according to experimental data [55].

The stimulus corresponding to the local metabolic conditions (S_m_) was computed assuming a metabolic signal substance added to flowing blood in each vessel in proportion to (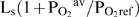) where L_s_ is the vessel length and 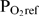 is the reference level for 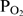 [56]. At each location within the vascular tree, the metabolic stimulus depends on the intravascular flux of the metabolite (J_m_) relative to the blood flow rate (Q) as S_m_ = log[1+J_m_/(Q+Q_ref_)] where Q_ref_, the reference value for blood flow, is a small constant included to avoid singular behavior at low blood flow values.

The convection of the metabolic substance with blood flow leads to downstream transmission of information about the metabolic state. In addition, a signal (J_c_) originating in each segment in proportion to the local value of the metabolic stimulus (S_m_) is assumed to be conducted upstream along the vessel wall. At branch points, the sum of the conducted stimuli from the draining segments is distributed evenly to the segments feeding into this branch point. The corresponding conducted stimulus (S_c_) depends on the value of J_c_ with a saturable response, S_c_ = J_c_/(J_c_+J_0_), where J_0_ is a reference value.

In previous studies of the same mesenteric microvascular networks [56], the parameters J_0_, 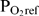, Q_ref_, k_p_, k_m_, k_c_ and k_s_ were estimated by minimizing the deviations between predicted and measured segment diameters and velocities using a multidimensional optimization procedure [57], yielding J_0_ = 27.9, 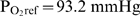, Q_ref_ = 1.98 nl/min, k_p_ = 0.7114, k_m_ = 0.6444, k_c_ = 2.113 and k_s_ = 1.69. In the present study, these values of J_0_, 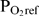 and Q_ref_ were retained. Simulations were carried out here for both tumor and mesentery networks using the same values of k_p_, k_m_, k_c_ and k_s_ and k_h_ = 1. This case was referred to as *deterministic adaptation*. In the previous studies [19],[56], k_h_ does not appear as a parameter in Eq. 3.

In considering the structure of tumor and mesenteric networks, it became evident that more detailed consideration of effects of experimental errors in diameter measurement and of inherent variability of biological responses was needed. In particular, a high degree of biological variability is apparent in tumor networks. To avoid biasing the results, it was necessary to consider the effects of such variability also in mesentery networks. First, effects of experimental errors in diameter measurement were considered. For a given network, a simulated adaptation with a given set of adaptation parameters was performed. Then, the characteristic network properties (O_2def_, d^3^_var_, V_tot_, ΔP) were calculated after randomly perturbing the obtained vessel diameters obtained from the simulated adaptation with an assumed mean RMS diameter measurement error of 1.02 µm according to experimental estimates for the intravital microscopy setting used [58]. The same mean RMS error was assumed also for the tumor network. The network characteristics reported here correspond to the median of 40 individual diameter randomization runs. The median was chosen since the observed distributions of the network characteristics appeared to be significantly skewed.

Next, the inherent variability of biological responses to a given level of stimulation (‘biological randomness’), was taken into account by randomizing the shrinking tendency, k_s_, in the simulated adaptation by adding for each segment a constant drawn from a normal distribution with mean 0 and a varying standard deviation (Ran-k_s_). This parameter had not been considered in our previous models. For each parameter setting, 25 simulated adaptations were done with values of k_s_ chosen from this distribution, with 40 individual diameter randomization runs for each k_s_ value. The median values from the randomized runs are reported for global network characteristics. The actual RMS measurement error for the tumor network may have been larger than 1.02 µm. In that case, the Ran-k_s_ value estimated for the tumor (0.23) would be an overestimate.

The sensitivity parameters k_h_, k_p_, k_c_, k_s_ and the randomization parameter Ran-k_s_ were varied to simulate the effects of different adaptation modes, holding k_m_ constant. Specifically, adaptation modes were sought that generate network characteristics similar to those found experimentally for the mesenteric and tumor microvessel networks. To establish these ‘normal’ and ‘tumor’ adaptation modes, the parameters were optimized to minimize the difference between the global network characteristics (O_2def_, d^3^_var_, V_tot_, ΔP) obtained after simulated adaptation and network characteristics obtained with measured vessel diameters for the mesenteric and tumor networks.

The choice of parameters to be estimated (k_h_, k_c_, k_s_, Ran-k_s_) was made according to the hypothesis that the vascular adaptation properties in tumors differ from normal terminal vascular beds. Based on the aberrant morphology and wall structure of tumor microvessels, it is plausible that such vessels exhibit altered sensitivity to hemodynamic forces of shear stress and pressure and a reduced capability to conduct electrical signals. In the simulated adaptation, these properties are controlled by the parameters k_h_ and k_c_, respectively. Also, the overall growth tendency within a tumor may be higher than in normal tissues due to a higher expression of vascular growth factors (e.g. VEGF) which is represented by a reduction of the shrinking tendency, k_s_. Inclusion of Ran-k_s_ allowed investigation of the contribution of intrinsic biological randomness to the differences between mesenteric and tumor networks. With this choice, the parameter k_h_ was no longer fixed to 1, as previously assumed. However, as already noted, the model predictions depend only on the ratios of k_h_, k_m_ and k_s_ and not on their absolute values. In practice, estimated values of k_h_ were close to 1, indicating that the overall balance between hemodynamic and metabolic sensitivities did not vary significantly between normal and tumor tissues.

### Optimization of model parameters for tumor and mesentery networks

Four different settings were used to test whether changed vascular adaptation may explain structural and functional differences between normal (N) and tumor (T) microvascular networks: optimization of the tumor network for experimentally observed tumor network characteristics (T/T) and for mesenteric network characteristics (T/N), and optimization of the mesenteric networks for experimentally observed mesenteric network characteristics (N/N) as well as for tumor network characteristics (N/T).

The target values for the optimizations for O_2def_, d^3^_var_, and ΔP were either the respective values for the tumor network (T: O_2def_ = 0.182, d^3^_var_ = 0.404, ΔP = 10.03 mmHg) or the mean value for the three mesenteric networks (N: O_2def_ = 0.0989, d^3^_var_ = 0.278, ΔP = 49.8 mmHg) obtained with measured vessel diameters. The target values for V_tot_ were chosen to reflect the different size of the networks considered: For T/T and N/N, the respective values for the individual networks were used (T: 6.59 nl; N: 43.54 nl, 24.75 nl and 20.78 nl for the three networks investigated). For T/N, the value obtained for the tumor network with deterministic adaptation was employed (1.58 nl) and the relation between this value and the value for the tumor with measured vessel diameters (6.59/1.58 = 4.17) was used to adjust target values for V_tot_ for N/T (190.12 nl, 99.69 nl and 95.5 nl).

**Figure 7:**
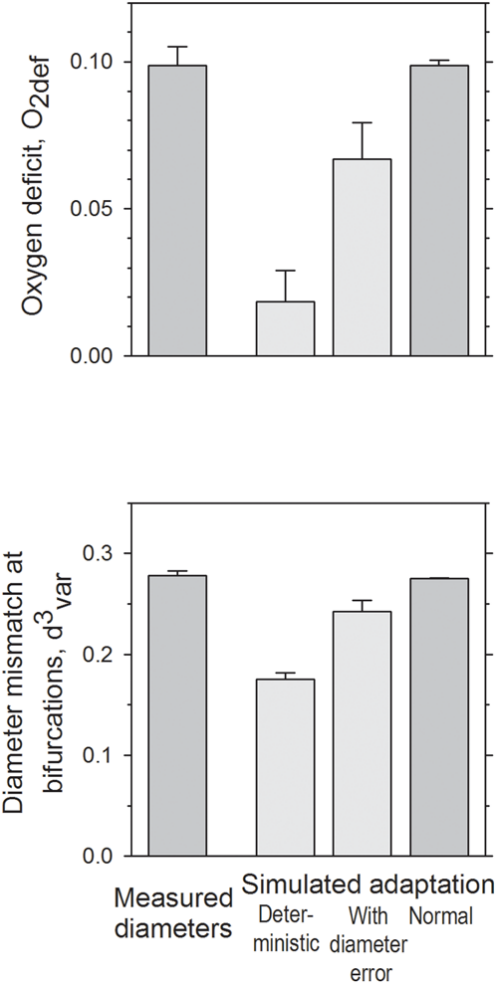
Functional and structural parameters for mesenteric networks obtained using measured vessel diameters and after simulated adaptation. Upper panel: oxygen deficit, O_2def_. Lower panel: variability of cubed diameter at bifurcations, d^3^_var_. Three different adaptation modes are shown. The ‘*deterministic adaptation*’ (second bars from left) uses sensitivity parameters as previously established for mesenteric networks [56] without considering diameter measurement error or biological heterogeneity. Levels for oxygen deficit and structural heterogeneity are lower than those using measured diameters. Inclusion of diameter measurement error brings the parameters close to those obtained with experimentally observed vessel diameters (third bars from left). The further addition of a biological variability in vascular sensitivity to local stimuli and optimization of sensitivity parameters (k_h_, k_c_, k_s_ and Ran-k_s_) allows for a close match of the simulation results to the experimental situation (*normal adaptation*, right bars). Mean values for three networks are shown with standard deviations.

The effects of these optimization steps on indices of network function and structure are illustrated in Figure 7. Deterministic adaptation leads to values for O_2def_ and d^3^_var_ substantially lower than those observed using measured vessel diameters. Inclusion of the inevitable effects of errors in the experimental determination of vessel diameters accounts for the majority of the observed discrepancy. A complete match could be achieved by including ‘biological randomness’ in the biological responses to a given stimulus, using the parameter Ran-k_s_ and optimizing the parameters k_h_, k_c_ and k_s_. The differences in k_h_, k_c_ and k_s_ for the optimized setting for normal adaptation relative to the deterministic adaptation were minor (k_h_ = 1.06±0.03 versus 1.00; k_c_ = 2.46±0.61 versus 2.11; k_s_ = 1.85±0.06 versus 1.69) while the optimal value for Ran-k_s_ was 0.10±0.02.
